# Genome-wide scan for runs of homozygosity identifies potential candidate genes associated with local adaptation in Valle del Belice sheep

**DOI:** 10.1186/s12711-017-0360-z

**Published:** 2017-11-14

**Authors:** Salvatore Mastrangelo, Marco Tolone, Maria T. Sardina, Gianluca Sottile, Anna M. Sutera, Rosalia Di Gerlando, Baldassare Portolano

**Affiliations:** 1 Dipartimento Scienze Agrarie, Alimentari e Forestali, Università degli Studi di Palermo, 90128 Palermo, Italy; 2 Dipartimento di Scienze Economiche, Aziendali e Statistiche, Università degli Studi di Palermo, 90128 Palermo, Italy

## Abstract

**Background:**

Because very large numbers of single nucleotide polymorphisms (SNPs) are now available throughout the genome, they are particularly suitable for the detection of genomic regions where a reduction in heterozygosity has occurred and they offer new opportunities to improve the accuracy of inbreeding ($F$) estimates. Runs of homozygosity (ROH) are contiguous lengths of homozygous segments of the genome where the two haplotypes inherited from the parents are identical. Here, we investigated the occurrence and distribution of ROH using a medium-dense SNP panel to characterize autozygosity in 516 Valle del Belice sheep and to identify the genomic regions with high ROH frequencies.

**Results:**

We identified 11,629 ROH and all individuals displayed at least one ROH longer than 1 Mb. The mean value of $F$ estimated from ROH longer than1 Mb was 0.084 ± 0.061. ROH that were shorter than 10 Mb predominated. The highest and lowest coverages of *Ovis aries* chromosomes (OAR) by ROH were on OAR24 and OAR1, respectively. The number of ROH per chromosome length displayed a specific pattern, with higher values for the first three chromosomes. Both number of ROH and length of the genome covered by ROH varied considerably between animals. Two hundred and thirty-nine SNPs were considered as candidate markers that may be under directional selection and we identified 107 potential candidate genes. Six genomic regions located on six chromosomes, corresponding to ROH islands, are presented as hotspots of autozygosity, which frequently coincided with regions of medium recombination rate. According to the KEGG database, most of these genes were involved in multiple signaling and signal transduction pathways in a wide variety of cellular and biochemical processes. A genome scan revealed the presence of ROH islands in genomic regions that harbor candidate genes for selection in response to environmental stress and which underlie local adaptation.

**Conclusions:**

These results suggest that natural selection has, at least partially, a role in shaping the genome of Valle del Belice sheep and that ROH in the ovine genome may help to detect genomic regions involved in the determinism of traits under selection.

**Electronic supplementary material:**

The online version of this article (10.1186/s12711-017-0360-z) contains supplementary material, which is available to authorized users.

## Background

Autozygosity is the homozygous state of identical-by-descent (IBD) alleles, which can result from several phenomena such as genetic drift, population bottlenecks, mating of close relatives, natural and artificial selection [1, 2]. The increase in inbreeding ($F$) leads to different negative effects such as reduction in genetic variance, higher frequency of homozygous genotypes for deleterious alleles with reduction in individual performance (inbreeding depression) and lower population viability [3]. Therefore, since $F$ has been incriminated in reduced fitness, there is a growing interest in characterizing and monitoring autozygosity for an accurate estimation of $F$. Traditionally, $F$ is estimated based on pedigree information. The current availability of very large numbers of single nucleotide polymorphisms (SNPs) throughout the genome makes these markers particularly suitable for the detection of genomic regions where a reduction in heterozygosity has occurred and offers the opportunity to estimate $F$ more precisely at the genome level. In fact, an alternative approach for quantifying individual homozygosity that better reflects IBD is based on runs of homozygosity (ROH). ROH are contiguous lengths of homozygous segments of the genome where the two haplotypes inherited from the parents are identical [4]. These haplotypes are most likely identical because the parents inherited them from a common ancestor. Nowadays, among several alternative methods to estimate inbreeding, $F$ estimated from ROH ($F_{\text{ROH}}$) is considered as the most powerful and makes it possible to distinguish between recent and ancient inbreeding [2]. ROH may be used to identify regions that have an unfavorable effect on a phenotype when they are in the homozygous state [5], but also to detect associations between traits of economic interest and genes present in these regions [6]. Indeed, given the stochastic nature of recombination, the occurrence of ROH is highly heterogeneous across the genome, and hotspots of ROH across a large number of samples may be indicative of selective pressure [7], which leads to the fixation of favorable alleles in the population. Identification of genomic regions that display a reduced level of polymorphism or no polymorphism (selective sweeps) may indicate occurrence of recent selection and may help to detect QTL and candidate genes. ROH have been studied in humans [4], cattle [8–10], pigs [11, 12], but less commonly in other livestock species, such as sheep. Here, we investigated the occurrence and the distribution of ROH in Valle del Belice sheep using a medium-density SNP genotyping array, in order to characterize autozygosity and identify the genomic regions with high ROH frequencies, namely ROH islands or ROH hotspots.

## Methods

### Ethics statement

In this study, the procedures for which animal samples were collected followed the recommendation of directive 2010/63/EU.

### Samples, genotyping and data editing

Blood samples were collected from 516 individuals (502 females and 14 males) of the Valle del Belice breed. Genomic DNA was hybridized with the Illumina OvineSNP50 K BeadChip (Illumina Inc., San Diego, CA, USA), which includes 54,241 SNPs. Raw signal intensities were converted into genotype calls using the Illumina GenomeStudio Genotyping Module v1.0 software by applying a no-call threshold of 0.15.

Genotyping data were initially tested for quality using the above-mentioned software. Chromosomal coordinates for each SNP were obtained from the latest release of the ovine genome sequence assembly Oar_v4.0. SNPs were filtered to exclude loci assigned to unmapped contigs and to sex chromosomes. Moreover, quality controls included the following criteria, i.e. a call frequency higher than 0.95, a minor allele frequency (MAF) higher than 0.05, and a *P* value higher than 0.001 for Hardy–Weinberg equilibrium (HWE). SNPs that did not satisfy these criteria were excluded. Animals with more than 5% of missing SNPs were also removed from further analyses.

### Measure of runs of homozygosity

Runs of homozygosity (ROH) were estimated for each individual using PLINK [13]. No pruning was performed based on linkage disequilibrium (LD), but the minimum length of a ROH was set to 1 Mb to exclude short ROH that derived from LD. The following criteria to define ROH were used: (1) one missing SNP was allowed in a ROH and up to one possible heterozygous genotype; (2) the minimum number of SNPs that constituted the ROH ($l$) was calculated with the method proposed by Lencz et al. [14], to minimize the number of false positive ROH:

$$l = \frac{{log_{e} \alpha /n_{s} \times n_{i} }}{{log_{e} \left( {1 - het} \right)}},$$

where $\alpha$ is the percentage of false positive ROH (set to 0.05 in this study), $n_{s}$ is the number of SNPs per individual, $n_{i}$ is the number of individuals, $het$ is the heterozygosity across all SNPs; (3) a minimum density of one SNP over 100 kb; and (4) a maximum gap between consecutive SNPs of 1 Mb.

### Genetic diversity and genomic inbreeding coefficients

PLINK [13] was also used to estimate basic genetic diversity indices including observed and expected heterozygosity ($${\text{H}}_{\text{o}}$$ and $${\text{H}}_{\text{e}}$$, respectively), the inbreeding coefficient ($$F$$) based on the difference between the observed and expected numbers of homozygous genotypes, and minor allele frequency (MAF ≥ 0.05). The molecular coancestry coefficient (also called kinship) ($$f_{ij} )$$ between individuals $$i$$ and $$j$$ was also estimated [15]. Moreover, the contemporary effective population size ($${\text{N}}_{\text{e}}$$) was estimated using NEESTIMATOR v.2 [16] according to the random mating model of the LD method. Inbreeding coefficient ($$F$$) based on ROH ($$F_{\text{ROH}}$$) for each animal was calculated as:

$$F_{\text{ROH}} = \frac{{{\text{L}}_{\text{ROH}} }}{{{\text{L}}_{\text{aut}} }},$$

where ${\text{L}}_{\text{ROH}}$ is the total length of all ROH in the genome of an individual while ${\text{L}}_{\text{aut}}$ refers to the length of the autosomal genome covered by SNPs included in the array (2644.30 Mb). Pearson’s correlation between the two measures of inbreeding ($F$ and $F_{\text{ROH}}$) was calculated.

### Distribution of runs of homozygosity

The mean number of ROH per individual (${\text{MN}}_{\text{ROH}}$), the average length of ROH (${\text{AL}}_{\text{ROH}}$) and the total number of ROH per animal were estimated. The percentage of chromosomes covered by ROH was also calculated. First, the mean ROH length was calculated by summing all ROH (Mb) on a chromosome (OAR for *Ovies aries* chromosome) and dividing by the number of individuals that had ROH on that OAR; the mean ROH length was then divided by the length of the chromosome in Mb. In addition, chromosomal $F_{\text{ROH}}$ ($F_{\text{ROHOAR}}$) values were also estimated, as $F_{\text{ROHOAR}} = {\text{L}}_{\text{ROHOAR}} /{\text{L}}_{\text{OAR}}$, where ${\text{L}}_{\text{ROHOAR}}$ is the total length of an individual’s ROH for each OAR and ${\text{L}}_{\text{OAR}}$ is the length of each OAR covered by the SNPs involved.

### Detection of common runs of homozygosity

To identify the genomic regions that were most commonly associated with ROH, the percentage of the occurrences of a SNP in ROH was calculated by counting the number of times the SNP was detected in those ROH across individuals, and this was plotted against the position of the SNP along the chromosome. This percentage had to be higher than 20% to be an indication of a possible hotspot of ROH in the genome. A series of adjacent SNPs with a proportion of ROH occurrences higher than the 20% threshold formed long genomic regions, called ROH islands.

To verify if recombination rate affected ROH length, ROH were mapped using the genetic SNP coordinates (position in the linkage map) reported by Johnston et al. [17]. The average recombination rate (cM/Mb) was estimated in 500-kb intervals for the chromosomes with the highest *F*_ROHOAR_ values and also within each ROH hotspot, and the percentage of occurrences of a SNP in ROH was plotted against recombination rate for the aforementioned chromosomes. In addition, genetic mapping of ROH lengths was used to infer demographic events by applying the method proposed by Thompson et al. [18] and recently reported by Purfield et al. [19] in a study on meat sheep breeds, following the same four ROH length categories.

Genomic coordinates for all identified selected regions were used to annotate genes that were either entirely or partially included within each selected region using the Genome Data Viewer (https://www.ncbi.nlm.nih.gov/genome/gdv/browser/?context=gene&acc=101104604) provided by NCBI. The function of these genes and pathways in which they are involved were assessed using Panther software [20] and the Kyoto Encyclopedia of Genes and Genomes (KEGG, http://www.genome.jp/kegg/) database. Finally, to investigate the biological function of each annotated gene within ROH islands, we conducted an extensive accurate literature search.

## Results and discussion

Analysis of ROH based on genomic data can help to describe the history of the population to which an individual belongs and can also reveal the level of inbreeding within populations, recent population bottlenecks or signatures of directional selection. However, to date, literature on ovine ROH is scarce, although sheep represent excellent genetic resources that contribute to local economy. To the best of our knowledge, this study is the first effort to describe the occurrence and distribution of ROH in a large number of individuals using medium-density SNP arrays in dairy sheep.

### General statistics

After filtering, the final number of samples and SNPs retained for analyses were 497 and 38,815, respectively. The average observed (${\text{H}}_{\text{o}}$) and expected (${\text{H}}_{\text{e}}$) heterozygosities were 0.373 ± 0.118 and 0.377 ± 0.117, respectively, and the average MAF was 0.288 ± 0.128. These values were consistent with the range reported by other authors for southern European sheep [21] and in previous studies on this breed [22]. A low and positive $F$ (0.011 ± 0.073) was observed, which suggests that the sampled animals are not highly related [6]. This result was also confirmed by the kinship coefficients, which were positive between all pairs of animals (0.498 ± 0.060). The contemporary effective population size (${\text{N}}_{\text{e}}$) was about 45, which indicates a high risk of inbreeding and reduced genetic diversity. Recently, a study on Australian sheep breeds based on SNP data and using the same method [23] reported large ${\text{N}}_{\text{e}}$ for all investigated breeds (ranging from 140 to 348), whereas another study reported a small ${\text{N}}_{\text{e}}$ [25] for the Sicilian Barbaresca breed [24].

### Distribution of runs of homozygosity

Because strong LD, typically extending up to about 100 kb, is common throughout the ovine genome [22], short tracts of homozygosity are very prevalent. To exclude these short and very common ROH, the minimum length for ROH was set at 1 Mb, with a minimum number of 40 SNPs. Moreover, we estimated ROH longer than 1 Mb with one heterozygous SNP in order to avoid underestimation of long ROH. In fact, for livestock populations, not allowing for heterozygous SNP genotypes in a ROH, as was advocated for human populations, is not adequate because they have much higher levels of autozygosity and therefore longer ROH [25]. Moreover, because genotyping errors in SNP chip data can occur, it is more reasonable to allow one heterozygous call per ROH.

**Fig. 1:**
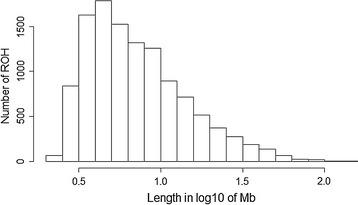
Distribution of the number of runs of homozygosity (ROH) of different lengths (Mb). The values of length in Mb were transformed in log_10_

In total, 11,629 ROH were identified with a MN_ROH_ of 24.20 ranging from 1 to 66 ROH per animal. Similar results were reported by Al-Mamun et al. [23]. All Valle del Belice individuals displayed at least one ROH longer than 1 Mb. The mean value of $F_{\text{ROH}}$ for ROH longer than 1 Mb was equal to 0.084 ± 0.061 and ranged from 0.002 to 0.339. The coefficient of variation (72.6%) indicated that autozygosity levels in this breed varied largely and the correlation between $F$ and $F_{\text{ROH}}$ was high (0.97, p value < 0.001). These results corroborate previous studies in cattle [10] and sheep [19]. For the three animals that had the highest level of homozygosity, respectively 807.85, 827.49, and 895.88 Mb of their genome were classified as ROH, which is close to 30% of the genome. The least inbred animal presented only one ROH of 4.95 Mb. An average ROH length of 9.51 Mb was estimated across all the autosomes but the lengths of ROH varied considerably ranging from 2.15 to 127.72 Mb. It is likely that the values for total ROH length and number reported in our work are underestimated because many ROH remain undetected when using a medium-density SNP panel [26]. The genomic distribution, length and abundance of ROH constitute a valuable source of information about the demographic history of livestock species [27]. The distribution of ROH according to size is in Fig. 1, in which the length in Mb was log-transformed. Our results show that ROH shorter than 10 Mb predominated. Because recombination events interrupt long chromosome segments, long ROH (~ 10 Mb) arise as a result of recent inbreeding (up to five generations ago). In contrast, short ROH (~ 1 Mb) are produced by IBD genomic regions from old ancestors and are indicative of more ancient relatedness (up to 50 generations ago) [28], which is frequently unaccounted for in the recorded pedigree of an individual.

Following a recent study on the distribution of ROH in sheep [19], we mapped ROH using their genetic positions and used the abundance of ROH in different length classes to qualitatively evaluate the historical demography of the breed. The time to the most recent common ancestor (TMRCA) was estimated for four different categories. The results show a substantial increase in the abundance of ROH from 10 to 20 generations ago to less than 5 generations ago (see Additional file 1: Figure. S1) in the Valle del Belice breed, which suggests a recent decrease in the effective population size, and that the individuals involved in our study experienced both recent and historical autozygosity events. Similar results were reported for the Belclare breed [19]. Therefore, it is likely that the long ROH detected in the Valle del Belice breed are signatures of the extended use of a few rams within herds and extensive mating between relatives. In fact, in the Sicilian farming system, natural mating is the common practice and the exchange of rams among flocks is quite unusual, which results in an increase in inbreeding within a herd and a consequent decrease in variability [29]. Indeed, the accumulation of long ROH in individuals could have consequences on the biological fitness. On the one hand, long ROH are enriched in genomic regions that carry deleterious mutations and there is a strong linear relationship between the genomic fraction of ROH and the number of individuals that carry deleterious homozygous mutations [30]. For example, a large number of genomic regions that contain long ROH were shown to have unfavorable associations with milk yield in Holstein cattle, probably because of inbreeding depression [5]. On the other hand, short ROH are subject to selection for a longer period of time and recombination has had more time to trim down ROH that are the target of selection sweeps [30]. In addition, it should be underlined that not all short ROH are due to IBD and it is possible that some short ROH originated from identity-by-status (IBS) due to localized low recombination rates, genetic drift and high LD in unrelated ancestors [19].

**Fig. 2:**
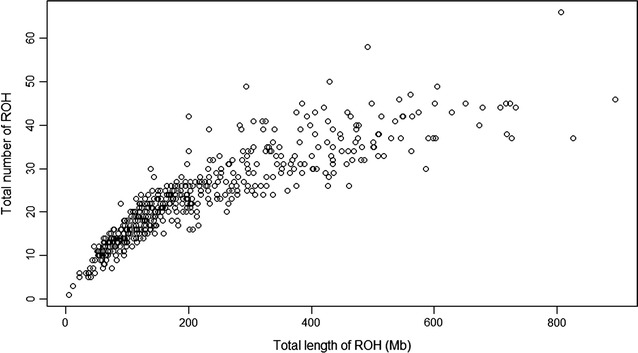
Total number of runs of homozygosity (ROH) longer than 1 Mb and total length of genome (Mb) covered by ROH segments per individual

The relationship between the number of ROH and the length of the genome covered by ROH per individual varies considerably among animals (see Fig. 2).

For example, animals with the same cumulative length of ROH can have a different number of ROH with different lengths, because of their different distances from the common ancestor [31]. As mentioned above, Valle del Belice sheep have a large number of short ROH with some extreme animals that have ROH that cover 600 Mb or more of the genome (Fig. 2). Similar distributions were also observed in other livestock species, such as cattle [10, 30] and pigs [32]. To identify outlying individuals more precisely, the observed total length of ROH was compared with a randomly generated distribution of ROH lengths among individuals. We carried out a simulation by sampling with replacement *n* values from the distribution of ROH (Fig. 1). We compared the observed total length of ROH to the simulated lengths and an empirical *p*-value was computed. The results are summarized in Figure S2 (see Additional file 2: Figure S2), in which some individuals are indicated in red because their *p*-values rejected the null hypothesis and thus, this highlights that some outlying individuals, with very long ROH, can be identified.

### Runs of homozygosity per chromosome

**Fig. 3:**
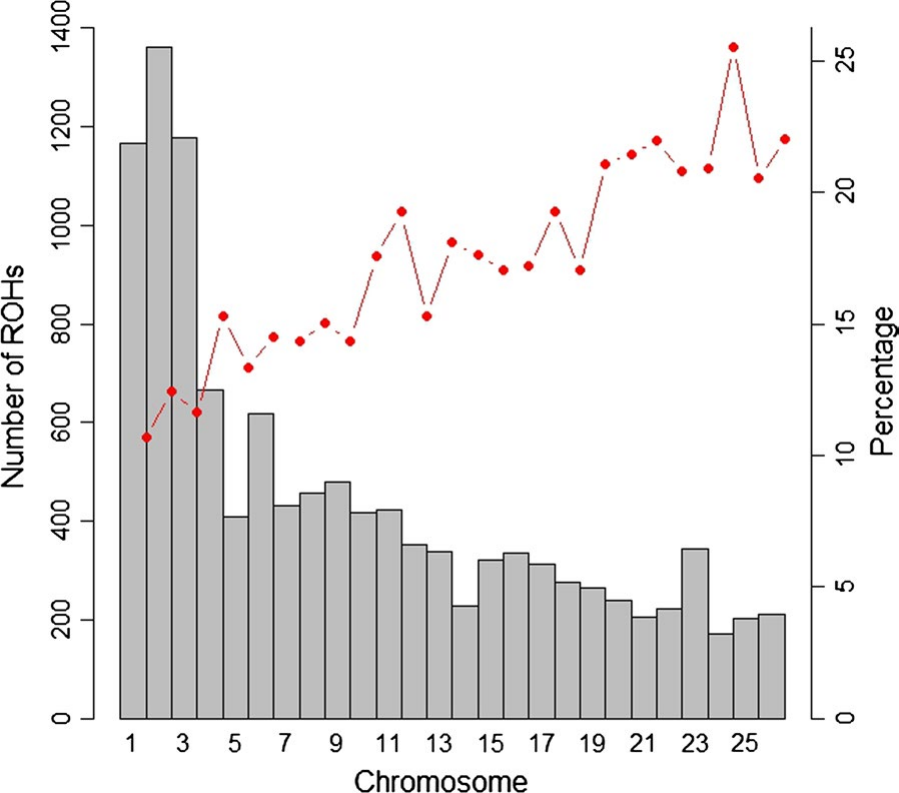
Number of runs of homozygosity (ROH) longer than 1 Mb per chromosome (bars) and average percentage of each chromosome covered by ROH (line)

Figure 3 shows the percentage of OAR chromosomes covered by ROH and the number of ROH per chromosome. The highest coverage by ROH was observed on OAR24 (25.54%), whereas the lowest was on OAR1 (10.68%). The number of ROH per chromosome displayed a specific pattern with the larger numbers found for the first three chromosomes, a number that tended to decrease with chromosome length, and the smallest number on OAR 24 with 172 segments. Regarding the number of ROH per OAR, our results confirm those reported in other sheep breeds [23], whereas for the percentage of coverage per chromosome, they are quite different which indicates that it may be breed-specific.

**Fig. 4:**
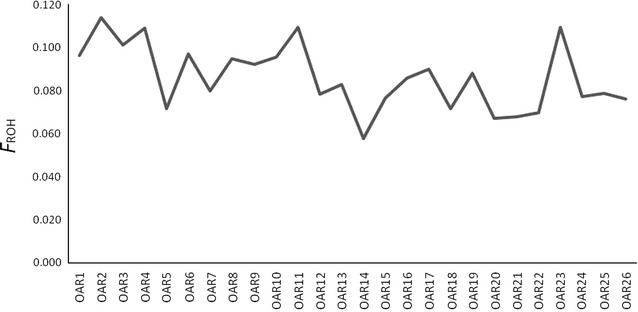
Distribution of inbreeding coefficients (*F*_ROHOAR_) based on runs of homozygosity (ROH) for each chromosome (OAR)

One of the main advantages of genomic coefficients is the availability of chromosomal inbreeding coefficients [10]. $F_{\text{ROHOAR}}$ estimates are reported in Fig. 4. In general, the mean $F_{\text{ROHOAR}}$ values followed the same pattern as those computed for the whole genome and differed between chromosomes. In particular, $F_{\text{ROHOAR}}$ values were highest for OAR2, 4, 11 and 23.

Genomic regions that are in close proximity to quantitative trait loci (QTL) subjected to selection are expected to show differences, such as reduced nucleotide diversity, and tend to generate ROH islands, with a high level of homozygosity around these genomic regions compared to the rest of the genome. In fact, significant QTL for milk production traits have been reported on these chromosomes in sheep i.e.: on OAR2, García-Gámez et al. [33] identified significant QTL for milk yield (ID = 57718 and ID = 57,737), milk protein (ID = 57719) and fat (ID = 57720 and ID = 57736) yields; on OAR4, QTL for milk protein yield [34] (ID = 57687) and milk fat percentage [33] (ID = 57721) were localized; on OAR11, Moioli et al. [35] reported QTL for milk fat (ID = 66000 and ID = 66001) and protein (ID = 66002) percentages, Jonas et al. [36] detected highly significant QTL for milk yield (ID = 16017), milk yield persistency (ID = 16018) and milk protein yield, whereas García-Fèrnandez et al. [37] reported QTL for fatty acid content (ID = 13896, ID = 13903, ID = 13904); and on OAR23, Gutiérrez-Gil et al. [38] identified significant QTL for milk yield (ID = 13906) and milk fat yield (ID = 13907) on OAR23. In Figures S3, S4, S5 and S6 (see Additional file 3: Figures S3, S4, S5 and S6), the occurrence of SNPs in ROH was plotted against the genomic regions of the above-mentioned QTL. These figures show that, except for OAR4, high levels of autozygosity on these chromosomes occur in the genomic region to which the QTL were mapped. Therefore, the differences in $F_{\text{ROHOAR}}$ patterns detected in our study may also highlight a specific effect of selection on these chromosomes for some milk production traits. However, it is also important to mention that the ROH identified in these genomic regions may be partly explained by a reduced recombination rate. Indeed, although the ROH are more or less distributed along the chromosome, ROH hotspots were mostly found in regions with a low recombination rate [19, 27]. In order to verify this distribution in the Valle del Belice breed, the occurrence of SNPs in ROH was plotted against the recombination rate for the aforementioned chromosomes with the highest $F_{\text{ROHOAR}}$ value (see Additional file 4: Figure S7). Figure S7 shows that the highest levels of autozygosity were often found within regions with a low recombination rate, as reported in a previous study [19]. However, genomic regions that harbor loci involved in general disease resistance, such as the major histocompatibility complex (MHC) for which a high level of genetic diversity ensures that the population can deal with potential new disease challenges, may show low levels of inbreeding [39]. In fact, the lowest values of $F_{\text{ROHOAR}}$ were reported for OAR14, on which significant genomic regions associated with resistance to nematode infection have been detected [40], and for OAR20, where the MHC is localized. These results indicate that genome-based measures of inbreeding through ROH are able to detect differences between chromosomal regions, providing a more detailed picture of the genetic diversity. Therefore, it might be possible to focus on the specific control of inbreeding on certain genomic regions because the level of diversity in those regions is already low (due to previous selection processes) or because they harbor genes for which populations with higher levels of diversity exhibit higher fitness (e.g. *MHC* genes) [41].

### Genomic regions within runs of homozygosity

**Fig. 5:**
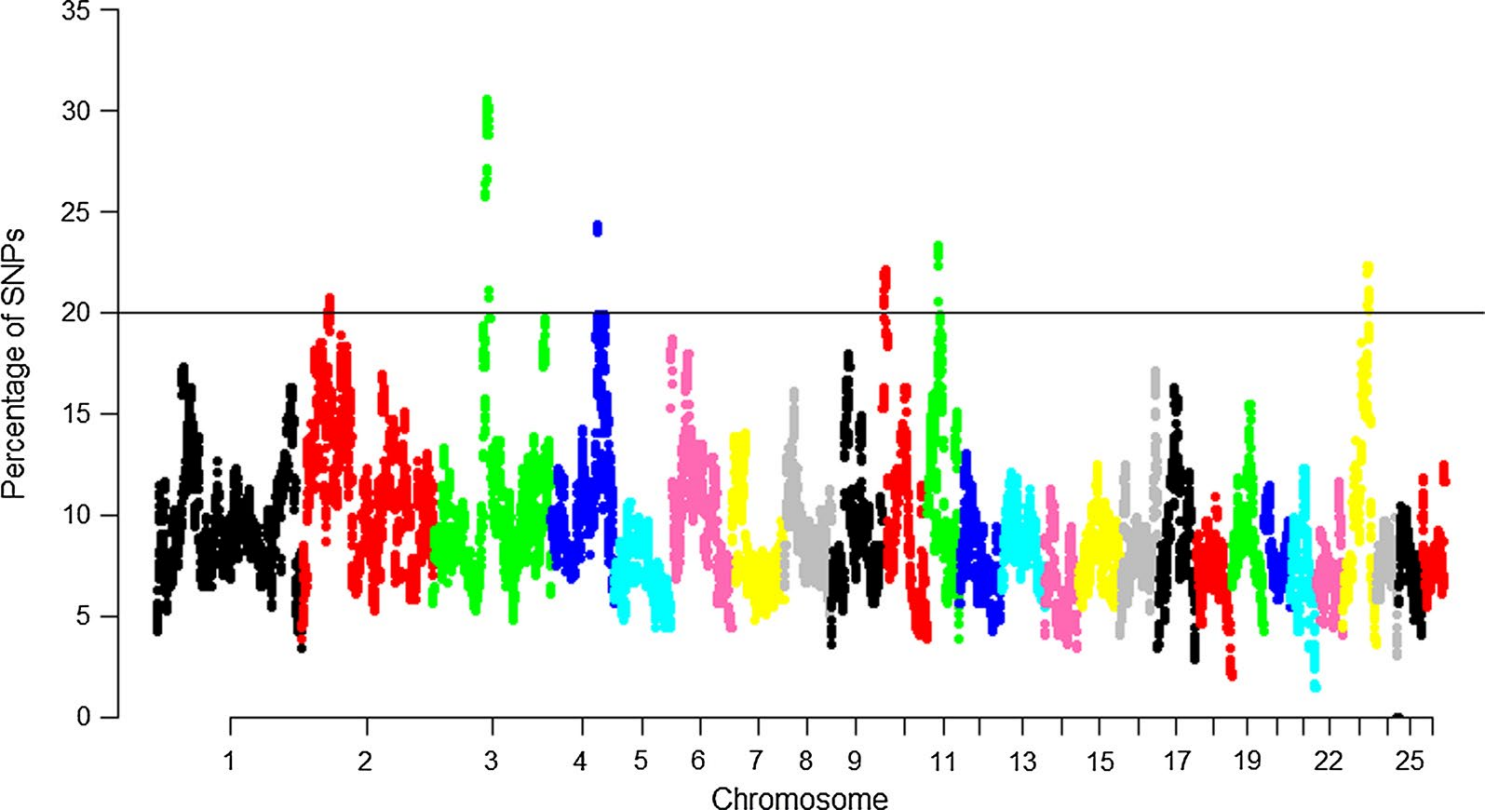
Manhattan plot of occurrences (%) of a SNP in ROH across individuals

To identify the genomic regions that were most commonly associated with ROH in the Valle del Belice breed, the percentage of SNPs in ROH was assessed by analyzing the frequency of a SNP occurring in those ROH across different individuals (%), and this was plotted against the position of the SNP along the chromosome (Fig. 5). The results show that ROH frequencies vary largely with the position on the genome. On OAR25, we found 37 non-consecutive SNPs that were not included in a ROH. Therefore, in this population, these SNPs are in the heterozygous state and indicate a region with a high level of heterozygosity. We set a threshold of 20% for considering a possible ROH hotspot in the genome. Two hundred and thirty-nine SNPs, i.e. less than 1% of all SNPs, were considered as candidate SNPs that may be under directional selection (see Additional file 5: Table S1). These adjacent SNPs were merged into genomic regions and considered as indicators of potential autozygosity islands, defined as genomic regions with extreme ROH frequency based on the Manhattan plot (Fig. 5). SNP rs411425463 on OAR3 was the most frequent SNP detected in ROH (152 occurrences). Six genomic regions located on six chromosomes (OAR2, 3, 4, 10, 11 and 23), presented hotspots of autozygosity (Table 1). The length of these regions ranged from 0.10 Mb on OAR4 to 6.60 Mb on OAR3. The region with the strongest signal was found on OAR3 and starts at position 99,745,362 bp (rs399921230) and ends at position 105,480,746 bp (rs399868290). Generally, as reported above, the existence of ROH hotspots can be partly explained by the reduced variation in recombination rate. Indeed, Purfield et al. [19] reported ROH hotspots within regions with very low recombination rates (from 0.00 to 0.82). The ROH hotspots reported in our study frequently coincided with regions with a higher recombination rate (from 0.47 to 1.64) (Table 1). Therefore, these results support the hypothesis that ROH patterns are not solely the result of demography and instead harbor targets of selection [19, 30].

**Table 1:** List of genomic regions of extended homozygosity detected in Valle del Belice sheep and average recombination rate (cM/Mb) within each hotspot

OAR	Start (bp)	End (bp)	Length (bp)	SNPs	Genes	cM/Mb
2	50,616,834	52,106,037	1,489,203	16	15	0.98
3	99,745,362	105,807,623	6,062,261	98	63	0.47
4	89,052,028	89,155,631	103,603	4	1	1.28
10	4,311,072	9,712,929	5,401,857	73	1	0.51
11	22,251,409	22,847,898	596,489	12	16	0.76
23	46,846,216	49,422,183	2,575,967	36	11	1.64

OAR = ovine chromosome, SNPs = number of SNPs in each genomic region, Genes = number of genes in each genomic region

We also checked if these regions of high homozygosity overlapped with putative selection signatures in sheep. We found that only the ROH on OAR2 partially overlapped with a region under climate-associated selection, which spanned two genes (*MELK* and *GNE*) [42] and with a selection signature detected in the Comisana breed [43]. Within all the genomic regions with a high level of autozygosity, we identified 107 genes (Table 1). Table S2 (see Additional file 6: Table S2) provides the chromosome position, start and end, full names and functions for all annotated genes. We found that several of the SNPs in ROH occurred in regions with few genes. In fact, some of the identified regions, such as that on OAR10, contain only one annotated gene, although it is longer than 5 Mb, either because annotation of the ovine genome is still incomplete or the genomic region is positioned outside a coding region. This result may also reflect selection acting on uncharacterized regulatory regions or simply fixation of non-coding DNA by genetic drift due to the absence of any selection [44]. According to Panther analysis and the KEGG database, most of the genes were involved in multiple signaling and signal transduction pathways of a wide variety of cellular and biochemical processes.

### Candidate genes within runs of homozygosity

In this paper, we do not discuss in detail all the genomic regions associated with ROH, but focus on some selected regions that show associations with several specific traits related to livestock breeding. Two genes were identified within the ROH on OAR2, i.e. *CLTA* associated with prion protein deposition in sheep [45] and *GNE*, which is important for the metabolism of sialylated oligosaccharides in bovine milk [46]. On OAR3, we identified an interesting gene, *NPAS2*, which encodes a protein that is part of the helix-loop-helix family of transcription factors, i.e. an essential component of the circadian clock [47]. The circadian clock, an internal timing system, regulates various physiological processes through the generation of about 24-h circadian rhythms of gene expression, which translate into metabolic and behavioral circadian rhythms. It acts as an important regulator of a wide range of physiological functions, including metabolism, body temperature, blood pressure, endocrine and immune functions [47]. Environmental variables such as photoperiod, heat, stress, nutrition and other external factors have profound effects on the quality and quantity of milk. How environment interacts with genotype to impact milk production is not known, but some evidence suggests that circadian clocks play an important role [47, 48]. Approximately 7% of the genes expressed during lactation have circadian patterns including core clock and metabolic genes [48]. Indeed, *NPAS2* was recently reported as a candidate gene for milk traits and SNPs in this gene are located within a reported QTL region for milk fat yield on chromosome 11 in cow [49]. Other putative candidate genes identified on OAR3 included: *ADRA2B* which plays an important role in vasoconstriction and blood pressure regulation [50], *PDCL3* for which the activity of its promoter was recently associated with heat stress [51], *LYG1* and *LYG2*, two *lysozyme g* genes (that encode an antibacterial enzyme) with an important role in innate immunity in vertebrate and non-vertebrate species [52], *CNGA3* which is associated with achromatopsia in sheep [53], *ANKRD23* and *ACOXL* which are involved in fatty acid and energy metabolism [54] and fat metabolism in pigs [55], respectively. On OAR11, we also identified *SERPINF1* and *SERPINF2*, which are involved in several biological and metabolic processes such as regulation of inflammatory response [56]. On OAR23, the ROH hotspot included the *endothelial lipase* (*LIPG*) gene, which plays a role in the reverse cholesterol transport (RCT) pathway that is a major component of lipid homeostasis affecting lipid phenotypes, such as different fat depots, fatty acid compositions and overall body growth [57]. *SMAD2* and *SMAD7* were also detected on OAR23, which code for a group of molecules that function as intracellular signal transducers downstream of the receptors of the TGF-β superfamily [58]. None of these candidate genes overlapped with previously published ovine QTL. Based on the literature search conducted for this study, only a few of the genomic regions that harbor candidate genes known to affect specific production traits were reported in previous studies in sheep. Moreover, in this study on the Valle del Belice breed, we did not identify important candidate genes for milk production traits in sheep in the detected ROH islands, such as the casein cluster, and the *DGAT1* or *ACACA* genes, which may be due to statistical or biological factors.

Generally, ROH patterns tend to differ between breeds [59]. Analysis of the distribution of ROH within a breed can provide insight into the effect of selection on the genome over varying periods of time and determine the direction of selection [30]. The Valle del Belice breed is subjected to limited breeding selection programs for milk production traits, but shows excellent adaptability to local environments, sometimes with harsh conditions. Thus, it is not surprising that the reported ROH islands spanned several candidate genes, which influence traits that are associated with adaptability in these environments and with the regulation of immune responses. In fact, our findings indicate that the genomic regions that display autozygosity in the Valle del Belice breed are mostly linked to selection in response to environmental stress as a result of local adaptation, and less to selection for milk production.

## Conclusions

In this study, we investigated the occurrence and the distribution of ROH in the genome of Valle del Belice sheep. Autozygosity levels varied largely in this breed, which has experienced both recent and historical inbreeding events. Several genes within ROH islands are associated with milk production and immune responses. These results suggest that natural selection has, at least partially, a role in shaping the genome of the Valle del Belice breed and that, in sheep, the analysis of ROH may contribute to detect genomic regions that are involved in the determinism of traits of economic importance.

## Additional files

**Additional file 1: Figure S1.** Mean sum of runs of homozygosity (ROH) per animal estimated within four different generation categories. ROH were mapped according to their genetic positions (i.e. linkage map positions). ROH length (l cM) within each category was determined using 100/2 *g*, replacing *g* with the number of generations of interest.

**Additional file 2: Figure S2.** Total number of runs of homozygosity (ROH) longer than 1 Mb and total length of genome (Mb) covered by ROH segments per individual. Observed (black) *vs* simulated (red) data.

**Additional file 3: Figures S3, S4, S5, S6.** Plot of SNP occurrences (%) in ROH against the genomic regions of QTL for OAR chromosomes with the highest inbreeding coefficient (OAR 2, 4, 11, 23).

**Additional file 4: Figure S7.** Plot of SNP occurrences in ROH against recombination rate. Recombination rate is the solid red line and the occurrence of a SNP in a ROH is represented by blue dots. (A) OAR2, (B) OAR4, (C) OAR11, and (D) OAR23. Recombination rate (cM/Mb) was estimated every 500 kb.

**Additional file 5: Table S1.** List of 239 SNPs considered as candidate markers under directional selection in the Valle del Belice sheep breed.

**Additional file 6: Table S2.** List of 107 potential candidate genes under directional selection in the Valle del Belice sheep breed.
