# Supplementary figures and images for: Genome-wide scan for runs of homozygosity identifies potential candidate genes associated with local adaptation in Valle del Belice sheep
Source: Genet Sel Evol. 2017 Nov 14;49:84. doi: 10.1186/s12711-017-0360-z (PMC5684758; doi:10.1186/s12711-017-0360-z)

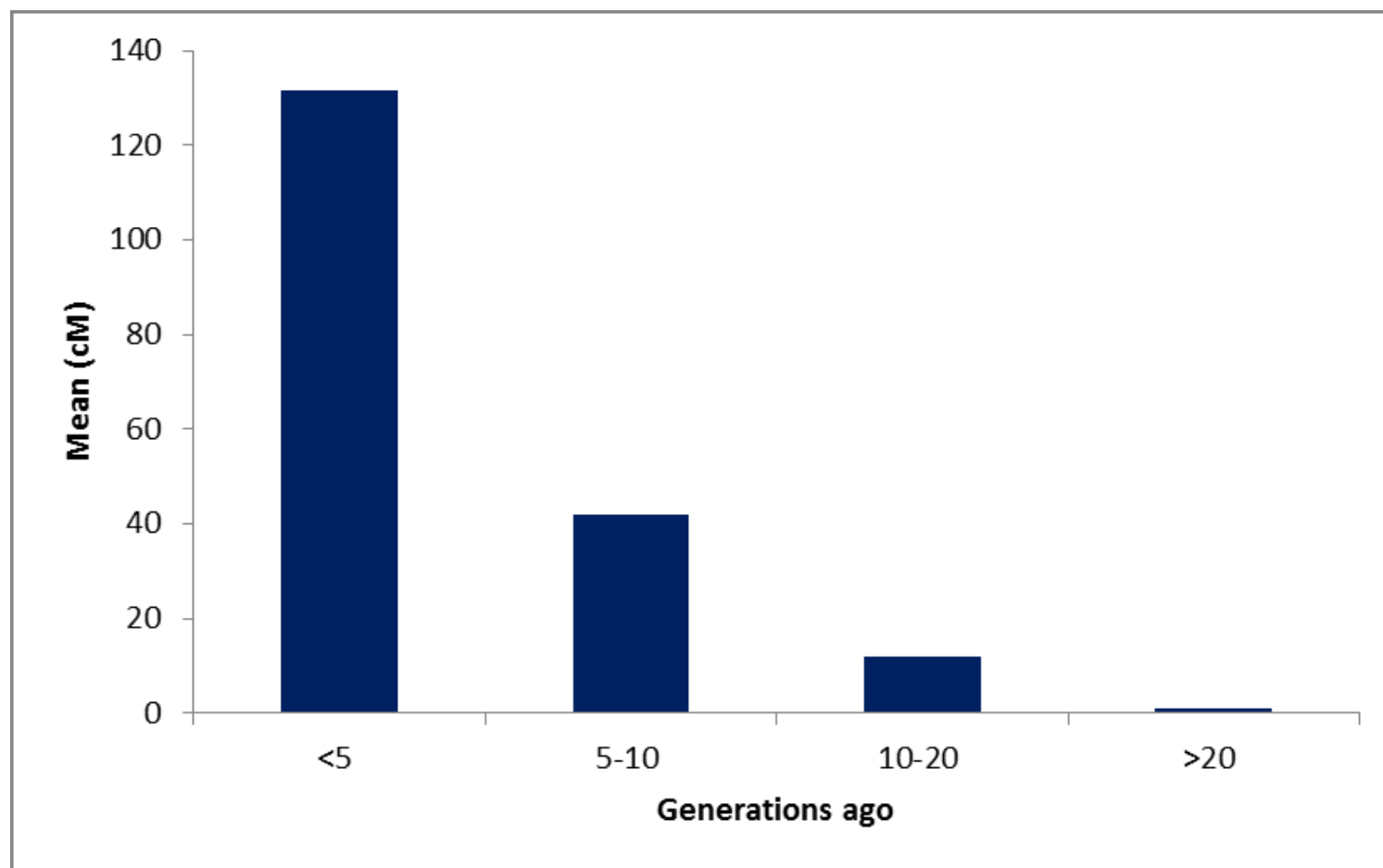

Supplement: Supplementary file 1 — Additional file 1: Figure S1. Mean sum of runs of homozygosity (ROH) per animal estimated within four different generation categories. ROH were mapped according to their genetic positions (i.e. linkage map positions). ROH length (l cM) within each category was determined using 100/2 g, replacing g with the number of generations of interest. [file 12711_2017_360_MOESM1_ESM.pdf]

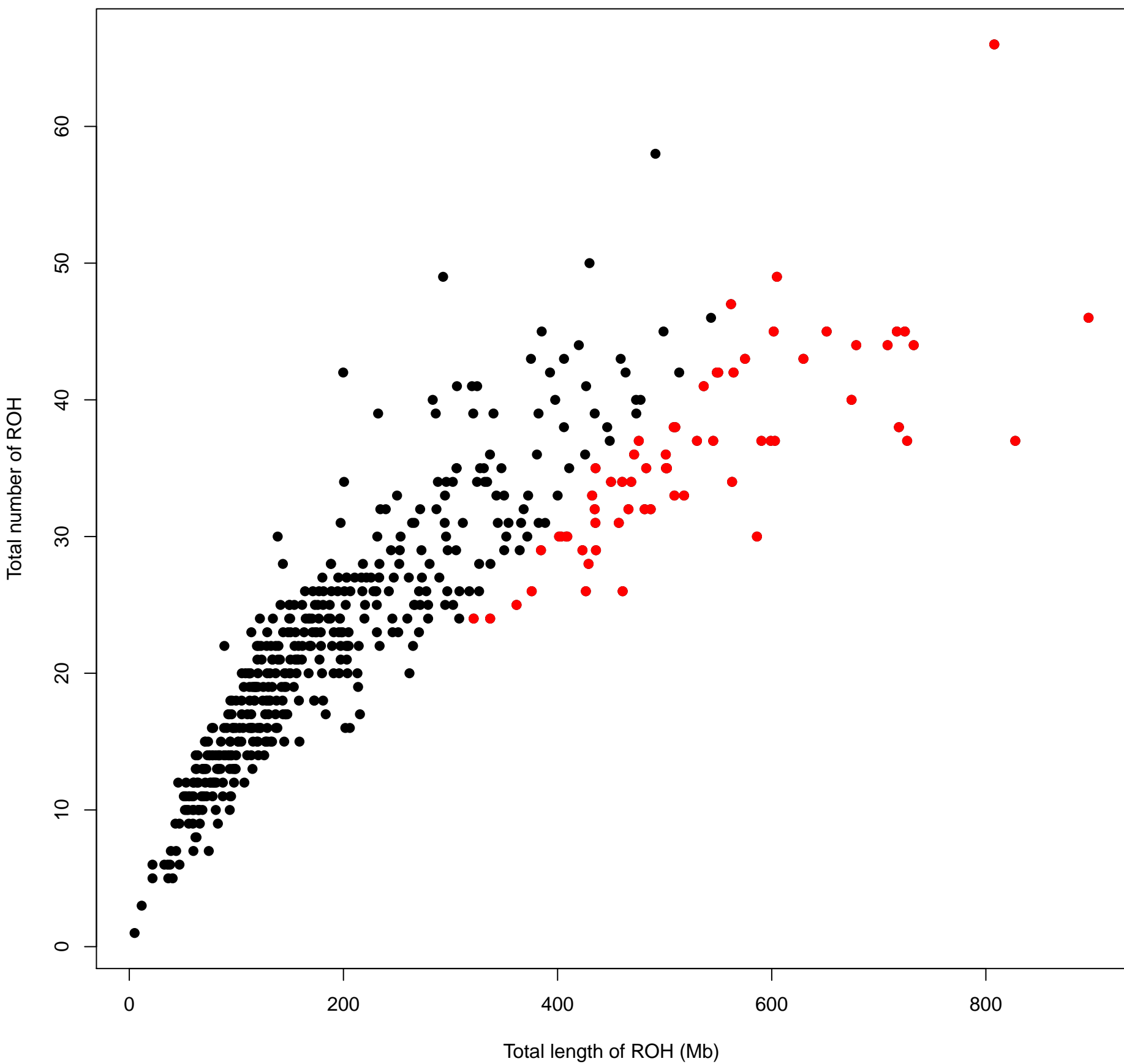

Supplement: Supplementary file 2 — Additional file 2: Figure S2. Total number of runs of homozygosity (ROH) longer than 1 Mb and total length of genome (Mb) covered by ROH segments per individual. Observed (black) vs simulated (red) data. [file 12711_2017_360_MOESM2_ESM.pdf]

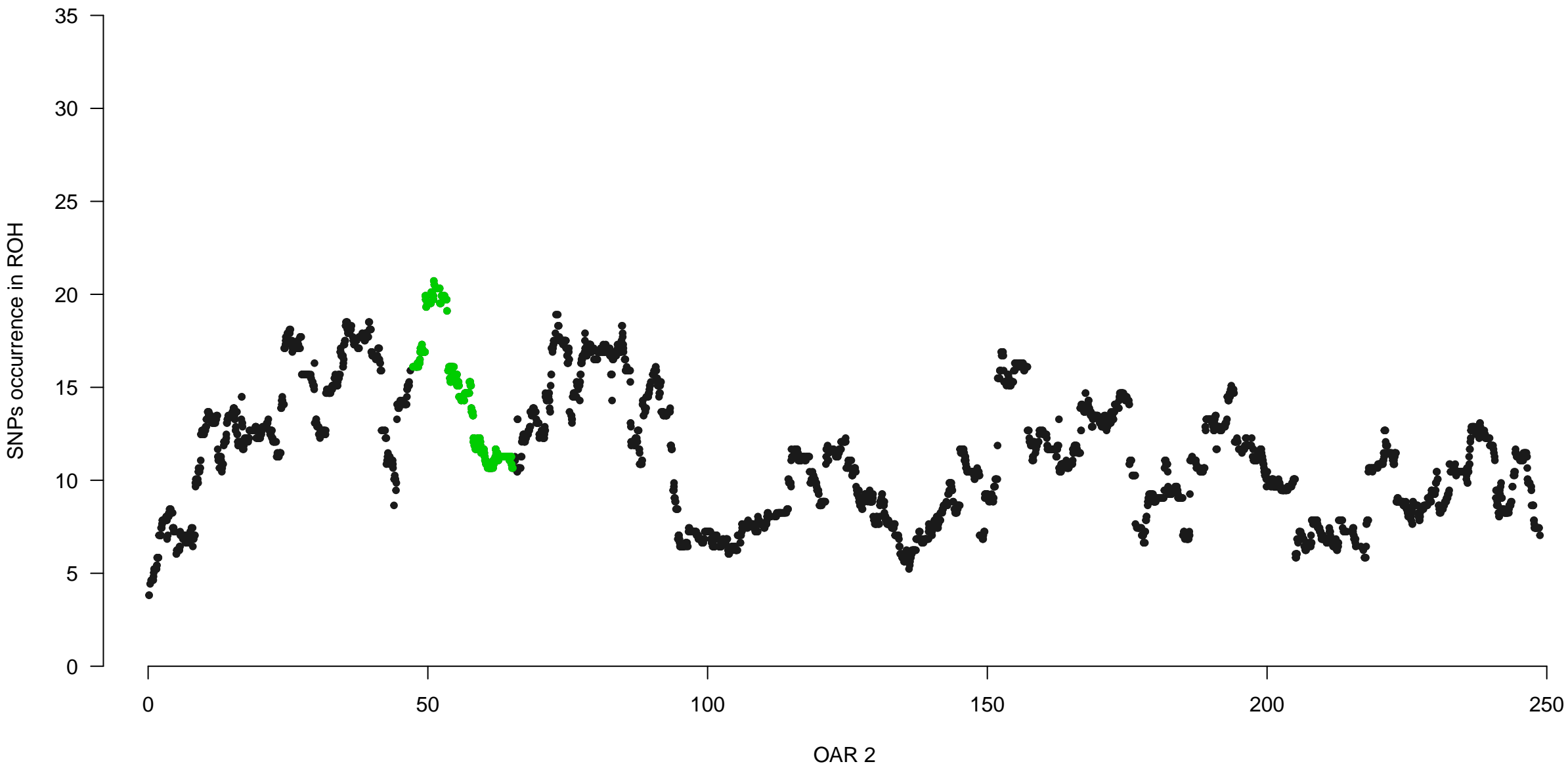

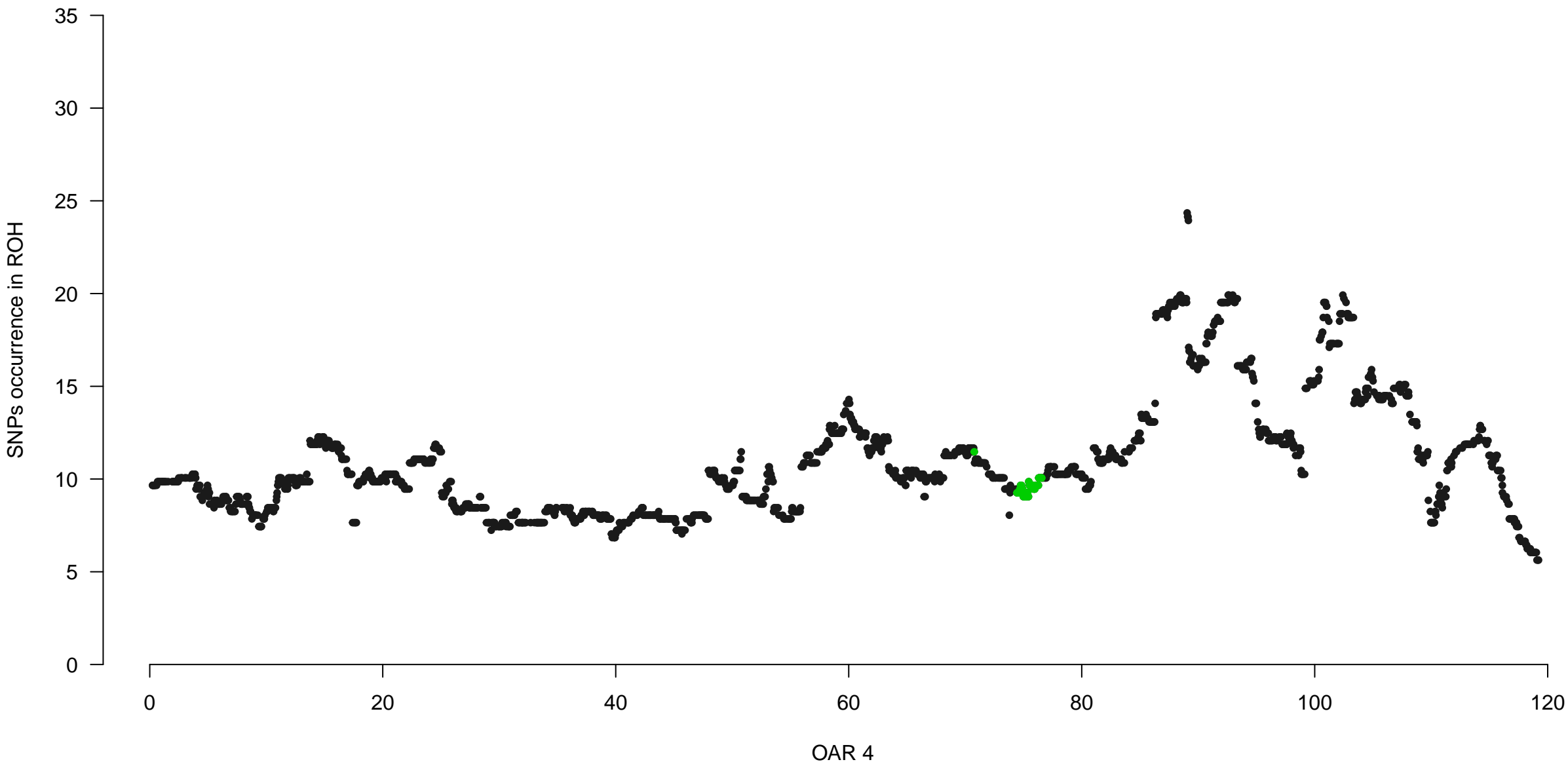

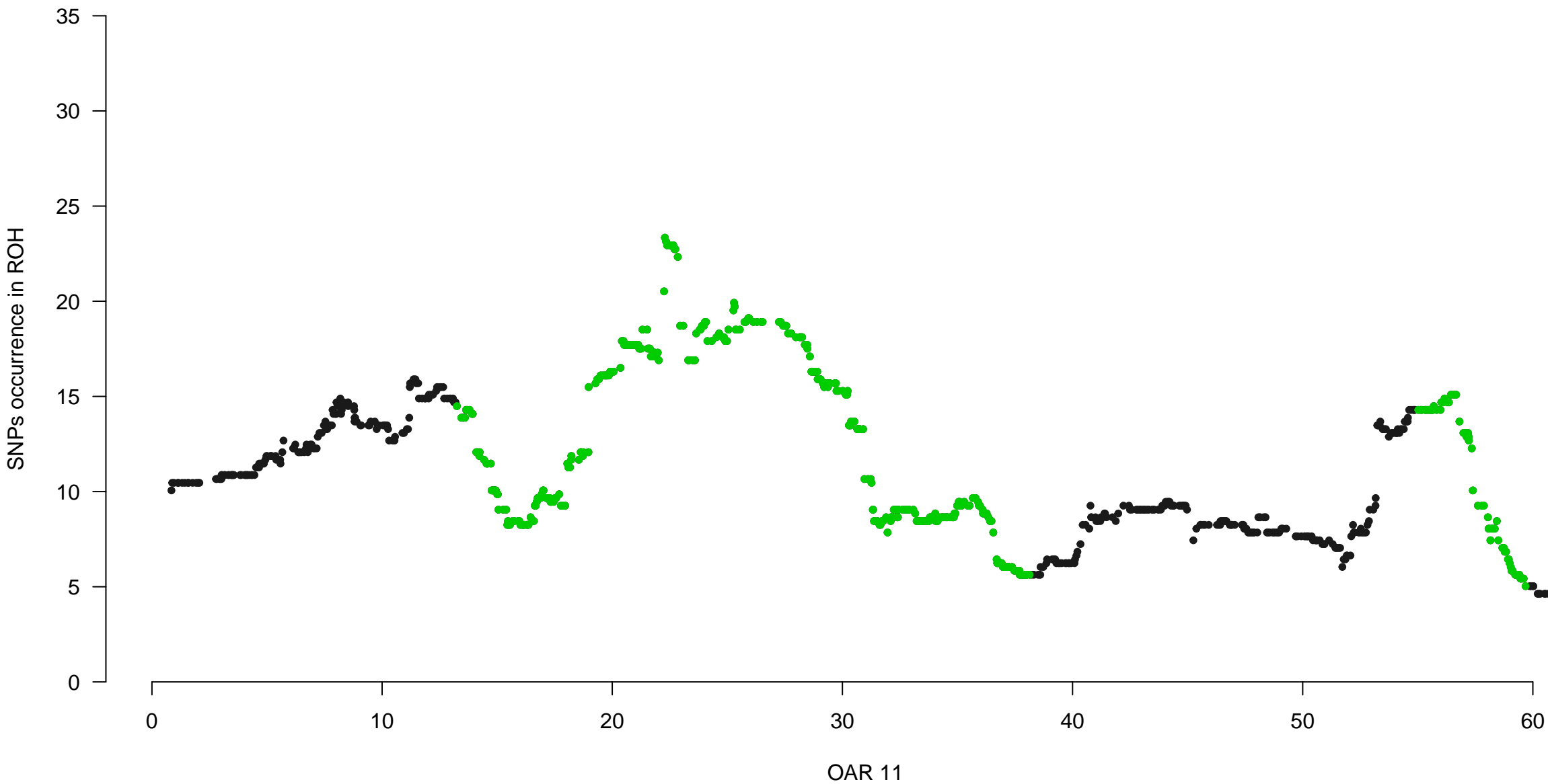

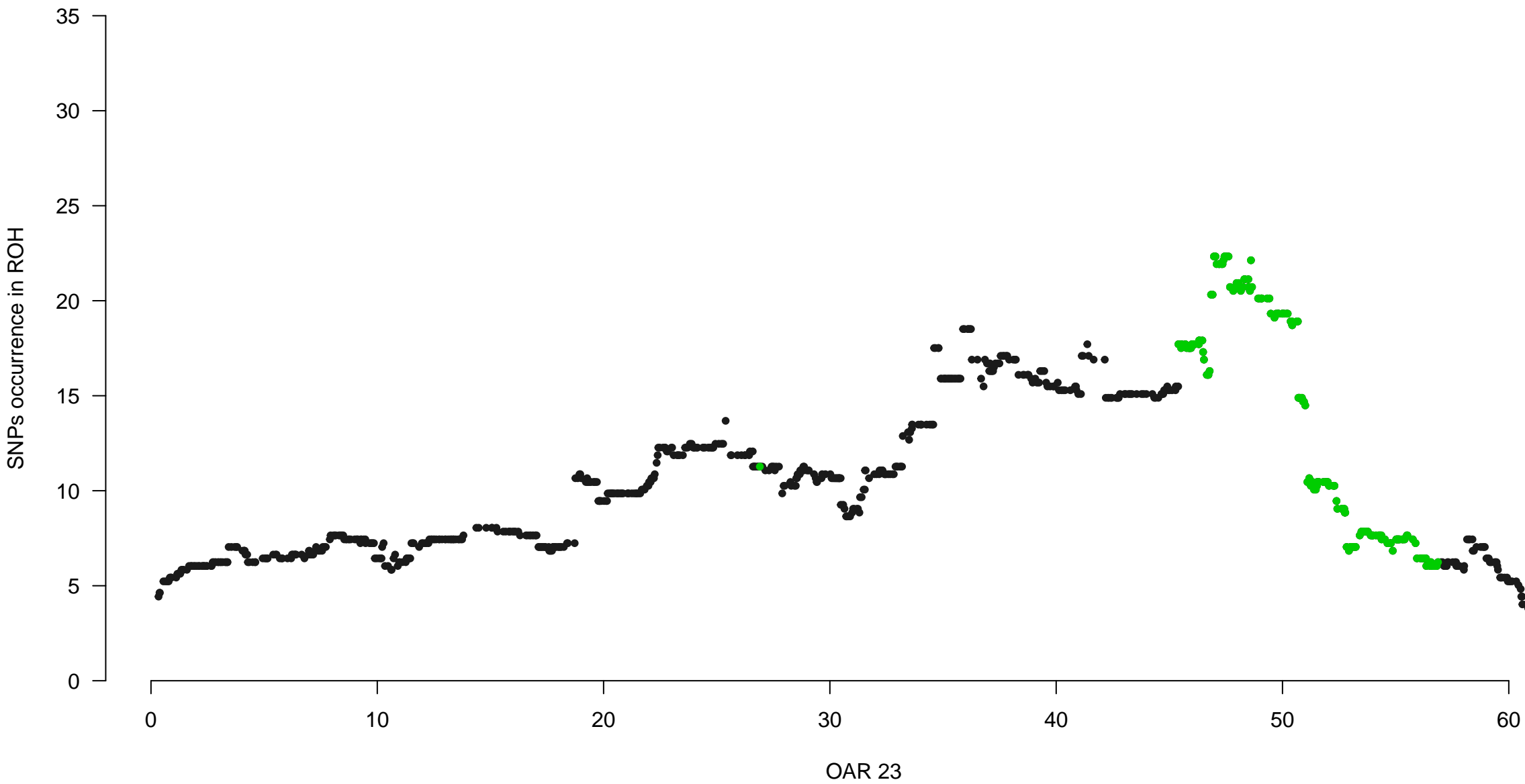

Supplement: Supplementary file 3 — Additional file 3: Figures S3, S4, S5, S6. Plot of SNP occurrences (%) in ROH against the genomic regions of QTL for OAR chromosomes with the highest inbreeding coefficient (OAR 2, 4, 11, 23). [file 12711_2017_360_MOESM3_ESM.pdf]

A)

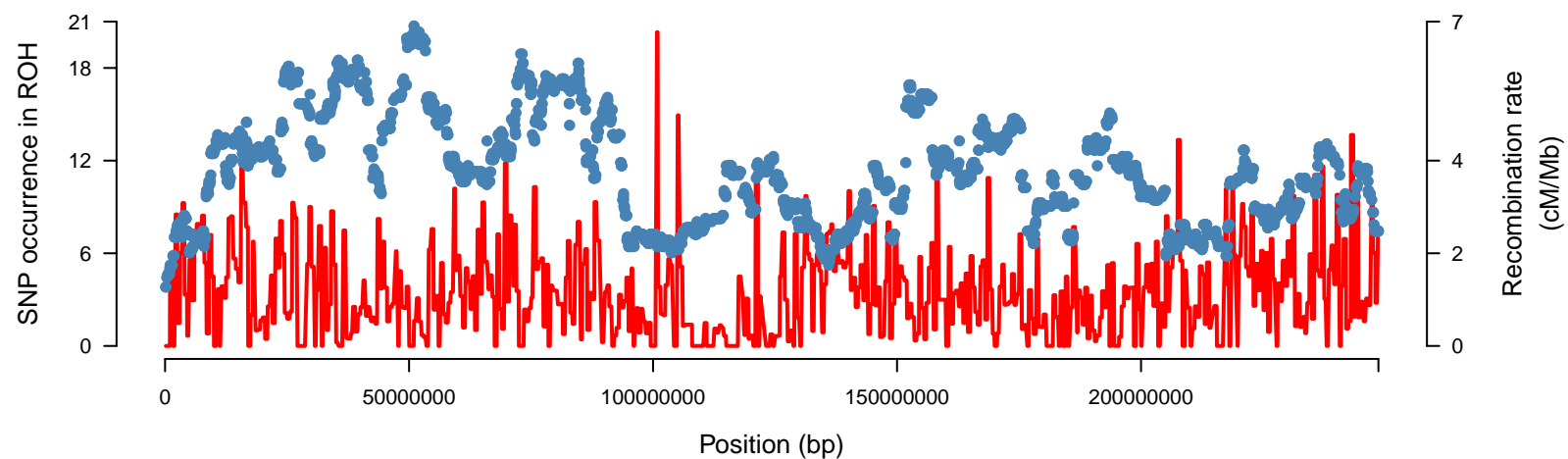

B)

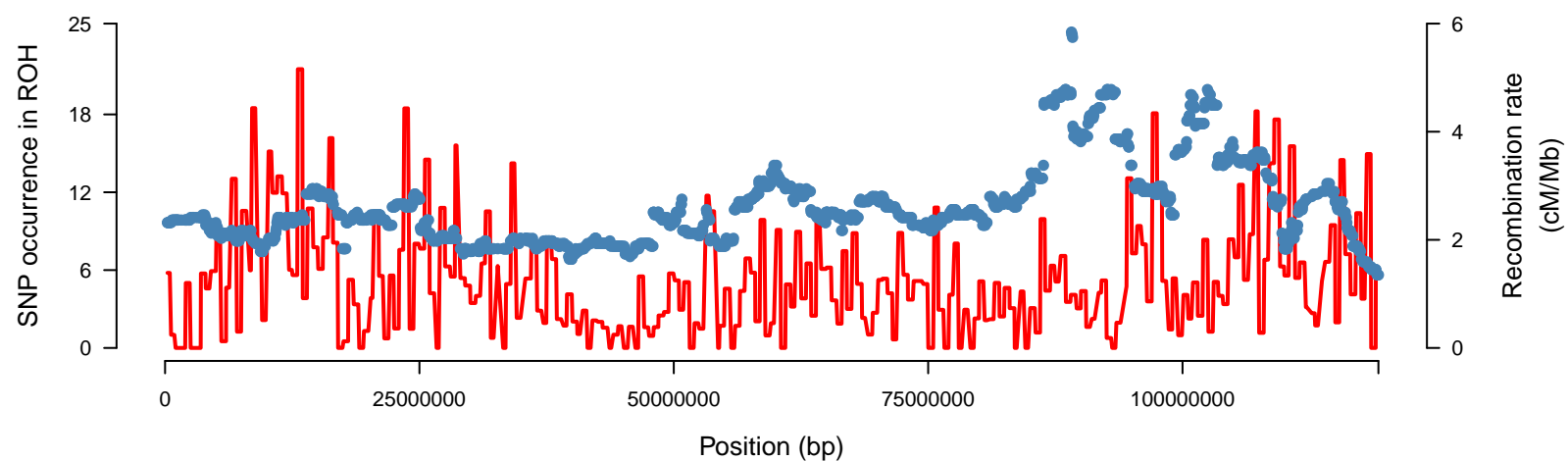

C)

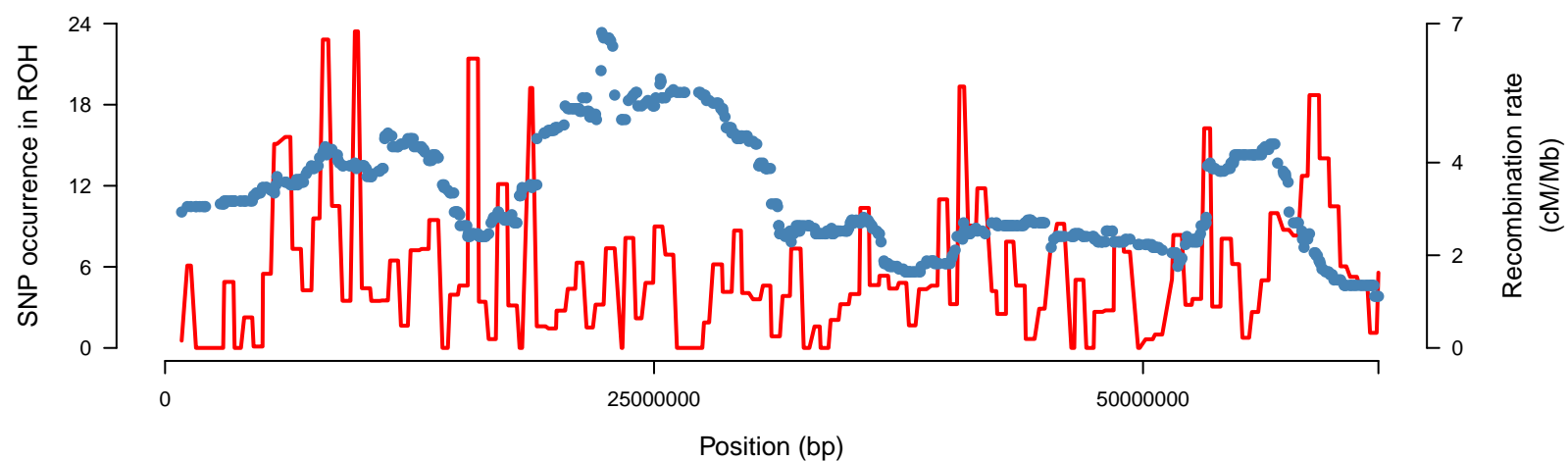

D)

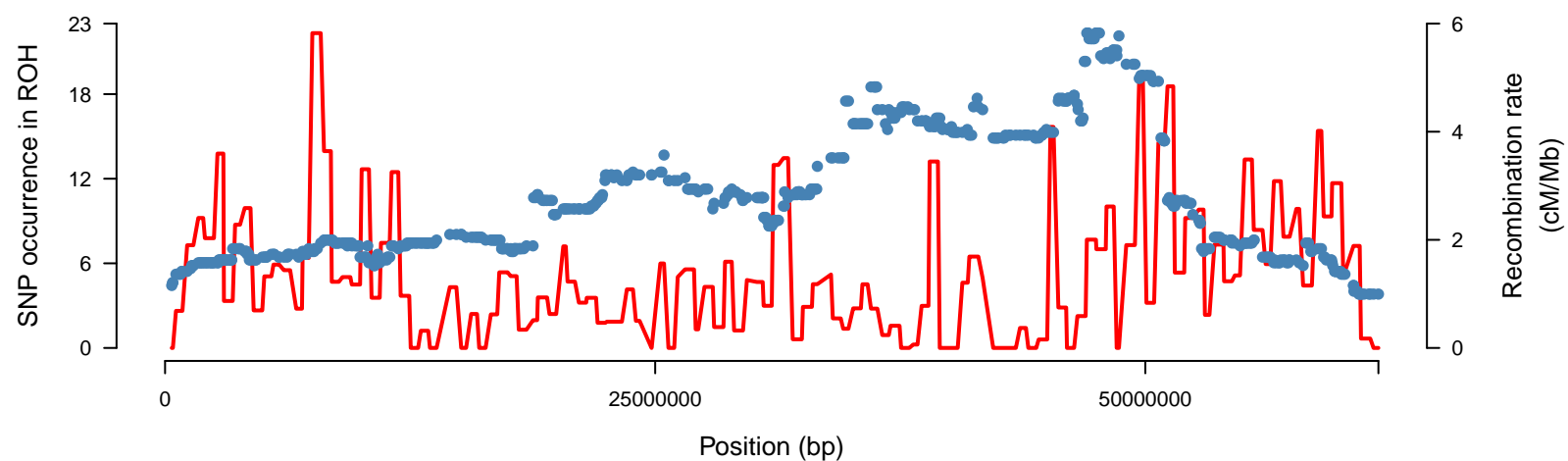

Supplement: Supplementary file 4 — Additional file 4: Figure S7. Plot of SNP occurrences in ROH against recombination rate. Recombination rate is the solid red line and the occurrence of a SNP in a ROH is represented by blue dots. (A) OAR2, (B) OAR4, (C) OAR11, and (D) OAR23. Recombination rate (cM/Mb) was estimated every 500 kb. [file 12711_2017_360_MOESM4_ESM.pdf]
